# Calcium potentiates the effect of estradiol on PGF2α production in the bovine endometrium

**DOI:** 10.1186/2049-1891-5-25

**Published:** 2014-05-05

**Authors:** Claudia Maria Bertan Membrive, Pauline Martins da Cunha, Flávio Vieira Meirelles, Mario Binelli

**Affiliations:** 1 São Paulo State University, Rod. Comandante João Ribeiro de Barros (SP 294) Km 651, Dracena, SP 17900-000, Brazil; 2 Department of Animal Reproduction, School of Veterinary Medicine and Animal Science, University of São Paulo, São Paulo, Brazil; 3 Department of Veterinary Medicine, School of Animal Sciences and Food Engineering, University of São Paulo, Pirassununga, Brazil

**Keywords:** Animal reproduction, Cattle, Estradiol, Luteolysis, PGF2α synthesis, Reproductive physiology

## Abstract

**Background:**

Estradiol (E_2_) is required for luteolysis in cows and its injection stimulates prostaglandin F2α (PGF2α) release. The main goal of our study was to investigate the ability of endometrial explants and cells treated with E_2_ and the calcium ionophore (CI) A23187 to synthesize PGF2α.

**Results:**

Treatment with E_2_*in vivo* resulted in a 48.4% increase of PGF2α production by endometrial explants treated *in vitro* with A23187. Production of PGF2α was better stimulated with A23187 at concentrations of 10^-6^ and 10^-5^ mol/L compared with other concentrations used. The concentration of PGF2α for untreated bovine endometrial cell cultures was 33.1 pg/mL, while for cultures treated with E_2_, A23187, or a combination of E_2_ and A23187, the PGF2α concentration was 32.5, 92.4 and 145.6 pg/mL, respectively.

**Conclusions:**

Treatment with A23187 tended to stimulate PGF2α production. In the presence of E2, A23187 significantly stimulated PGF2α synthesis. It appears that A23187 potentiates the effects of E_2_ with respect to synthesis of endometrial PGF2α in cattle.

## Background

In cattle, administration of 17β-estradiol (E2; 3 mg) as early as day 13 of the estrous cycle causes an increase in the plasma concentration of 13,14-dihydro-15-keto-prostaglandin F2α (PGFM), the main metabolite of PGF2α [1,2]. Consistently, ablation of ovarian follicles, which causes a decrease in plasma concentrations of E_2_, delays luteolysis in ruminants [3-5]. E_2_ is capable of inducing endometrial synthesis of PGF2α [6]; furthermore, E_2_ can induce luteolysis [7].

The endometrial synthesis of PGF2α results from a complex cascade of highly coordinated events. Arachidonic acid (AA), stored in the phospholipid membranes, is the primary precursor of prostaglandins [8]. Oxytocin (OT), acting through its receptor on the endometrial cell membrane, activates a guanosine nucleotide-binding protein (G protein), which promotes the activation of phospholipase C (PLC) [9]. PLC then cleaves phosphatidylinositol triphosphate into inositol triphosphate (IP_3_) and diacylglycerol (DAG). IP_3_ binds to receptors on the endoplasmic reticulum, promoting an increase in cytoplasmic calcium concentration. DAG activates protein kinase C (PKC), a serine/threonine kinase that is dependent on calcium for activation. Activated PKC phosphorylates phospholipase A_2_ (PLA_2_). The IP_3_-induced increase in cytosolic calcium stimulates calcium-dependent PLA_2_ activity [10]. PLA_2_ preferentially cleaves the sn-2 position of phosphatidylcholine, releasing AA [11]. The free AA is then converted into prostaglandin H_2_ (PGH_2_) by prostaglandin endoperoxide synthase 2 (PTGS2). Finally, PGH_2_ is converted into PGF2α by PGF synthases, such as Aldo-keto reductase family 1 member C1 (AKR1C1). Calcium is a known cofactor of PKC and PLA_2_, which are enzymes involved in PGF2α production. Although the proteins involved in the synthesis of PGF2α have been identified, the role of E_2_ in this process remains unknown.

The main goal of our study was to determine the ability of endometrial explants and cells to synthesize PGF2α. Specifically, we sought to evaluate the capacity of E_2_, when administered *in vivo*, to stimulate PGF2α synthesis in endometrial explants incubated with a calcium ionophore (CI), melittin or oxytocin. We also sought to determine the CI dose that was capable of stimulating the synthesis of PGF2α in cultured endometrial cells, and to evaluate the capacity of bovine endometrial (BEND) cells to synthesize PGF2α following treatment with E_2_ and/or a CI.

## Methods

### Experiment 1

We used 13 cyclic cross-bred beef heifers (*B. taurus* × *B. indicus*) in our study. Animals procedures were approved by Ethics and Animal Handling Committee of the Universidade de São Paulo. Animals were fed pasture (*Brachiaria decumbens* var. *marandu*) supplemented with minerals and had access to water *ad libitum*. Animals were implanted with a device containing 1 g of P4 (CIDR®, Pfizer, USA) along with an intramuscular injection of gonadorelin (100 μg; Fertagil®, Intervet, The Netherlands) on the first day of the synchronization protocol. Devices were removed 7 days later and cows were administered D-cloprostenol (150 μg; Preloban®, Intervet) intramuscularly and were marked on the tailhead using an All-Weather Paintstik® (LA-CO Industries Inc., USA). Estrous behavior was observed twice daily for 48–120 h after PGF2α was injected. The start of standing estrus was considered day 0 of the estrous cycle (day 0). On day 6, an ultrasonographic examination was performed (Aloka, SSD-500, linear probe 7.50 MHz), and females with a dominant follicle ≥7.5 mm received an intramuscular injection of gonadorelin (100 μg; Fertagil®, Intervet, The Netherlands) to induce ovulation of the dominant follicle and promote the emergence of a new follicular wave [12]. Ultrasonographic examination was performed to verify the presence of an accessory corpus luteum on day 16 of the cycle. Heifers were paired according to the day of standing estrus and randomly chosen for intravenous treatment with 0 (*n* = 6) or 3 mg of E_2_ (*n* = 7) on day 17. At 2 h post-treatment, animals were stunned by cerebral concussion from a pneumatic pistol and euthanized by jugular exsanguination. Genital tracts were transported to our laboratory on ice immediately after animals were euthanized. The endometrium of the ipsilateral horn to the original corpus luteum was dissected, and fragments of the intercaruncular region weighing 80–100 mg were conditioned in 12 mm × 75 mm borosilicate tubes containing 0.5 mL Krebs-Hensleit bicarbonate medium (KHB; 118 mmol/L NaCl, 4.7 mmol/L KCl, 2.56 mmol/L CaCl_2_, 1.13 mmol/L MgCl_2_, 25 mmol/L NaHCO_3_, 1.15 mmol/L NaH_2_PO_4_, 5.55 mmol/L glucose, 20 mmol/L Hepes and 0.013 mmol/L phenol red, pH 7.4). Cultures were maintained according to procedures described by Burns et al. [13]. Tubes containing explants were kept at 37°C in a shaking waterbath (40 rpm) for 1 h. Culture media was discarded and explants were washed twice with 0.5 mL of KHB. Explants were incubated for 1 h in KHB, washed, and then treated *in vitro* with 1 mL of either: KHB medium (control); KHB supplemented with 10^-5^ mol/L A23817, a CI (C-7522, Sigma Chemicals, USA); KHB supplemented with 10^-5^ mol/L melittin (M-2272, Sigma Chemicals, USA); or KHB supplemented with 10^-6^ mol/L OT (O-6379, Sigma Chemicals, USA). Explants from each cow received all treatments in triplicate. The concentrations of drugs we used were based on those previously reported [13-15]. Samples comprising 100 μL of culture medium were removed immediately after the administration of treatments (time 0) and 60 min later. Samples were stored at -20°C until required. The concentration of PGF2α (pg/mL/mg of endometrial tissue) in the culture medium was measured using radio immuno assays as described by Danet-Desnoyers et al. [14]. Intra-assay variation coefficients were 23.7 and 14.1%, and the inter-assay coefficients were 23.4 and 13.1% for standards containing 250 and 1,000 pg/mL PGF2α, respectively.

### Experiment 2

We obtained BEND cells [16] from the American Type Culture Collection (ATCC CRL-2398; USA). Cells were suspended in 4 mL of complete culture medium [40% HAM F-12 (N6760, Sigma Chemicals, USA); 40% minimal essential medium (MEM; M0643, Sigma Chemicals, USA); 200 IU/L insulin (I5500, Sigma Chemicals, USA); 10% (v/v) fetal bovine serum (FBS; 10270-106, Gibco Life, USA); 10% (v/v) equine serum (Nutricel, Brazil); and 1% (v/v) antibiotic and antimycotic solution (A7292, Sigma Chemicals, USA)] and seeded in 6-well tissue culture plates (Corning Incorporated, USA). Cells were cultured at 38.5°C/5% CO_2_ until 90% confluent. Subsequently, cells were maintained in serum-free medium for 24 h, washed twice in the same medium, and incubated for 12 h in 4 mL of serum-free medium supplemented with 0, 10^-7^, 10^-6^ or 10^-5^ mol/L A23817 (C-7522, Sigma Chemicals, USA) in triplicate wells. Samples of media (500 μL) were collected immediately after the administration of treatments (time 0) and 12 h later. After sample collection at time 0, the same volume of medium containing the specific treatment was replaced in culture wells. Samples were stored at –20°C until required. Experiments were repeated three times; intra-assay variation coefficients were 13.2 and 18.8%, and inter-assay variation coefficients were 10.3 and 22.0% for standards containing 250 and 1,000 pg/mL PGF2α, respectively.

### Experiment 3

We seeded BEND cells in 24-well plates (4 × 10^4^ cells/well; Corning Incorporated) with 1.5 mL of complete culture medium. Cultures were incubated at 38.5°C/5% CO_2_ until cells were 90% confluent. Cells were then cultured in serum-free medium for 24 h, washed twice with the same medium, and incubated for 12 h in serum-free medium supplemented with 10^-13^ mol/L E_2_ (E-8875, Sigma Chemicals, USA) and 10^-6^ mol/L A23817 (Sigma Chemicals, USA). Control wells received serum-free medium without supplements; all experiments were conducted in triplicate. Samples of media (300 μL) were collected immediately after the administration of drugs (time 0) and 12 h later. After sample collection at time 0, the same volume of medium containing the specific treatment was replaced in wells. Samples were stored at –20°C until subsequent measurement of PGF2α concentration. Experiments were conducted three times; intra-assay variation coefficients were 16.0 and 20.6%, and the inter-assay variation coefficients were 4.0 and 18.9% for standards containing 250 and 1,000 pg/mL PGF2α, respectively.

### Statistical analysis

Data that did not meet the assumptions of normality of residues (Shapiro-Wilk Test, *P* ≤ 0.01) or homogeneity of variances (F Test, *P* ≤ 0.01) were transformed by square roots and reanalyzed. Data were analyzed by ANOVA using the GLM procedure with the SAS program and are presented as untransformed least squares means ± SEM. Treatment means were compared by orthogonal contrasts. In Experiment 1, the dependent variable was DIF60 (the difference between PGF2α concentrations at 0 and 60 min), and the independent variable was treatment. For Experiments 2 and 3 the dependent variable was DIF12 (the difference between PGF2α concentrations at 0 and 12 h) and the independent variables were experiment, treatment and the interaction between experiment and treatment. Statistical significance was considered at *P* < 0.05.

## Results

### Experiment 1

The DIF60 for the various treatments are shown in Table 1. Endometrial explants that remained untreated *in vitro* produced similar concentrations of PGF2α whether they were obtained from cows injected with E_2_ (3 mg) (20.7 ± 4.2 pg/mL/mg of tissue) or not (24.7 ± 4.6 pg/mL/mg of tissue; Table 1). Regarding control animals, PGF2α synthesis was similar among untreated explants (24.7 ± 4.6 pg/mL/mg of tissue) and explants treated with OT (28.2 ± 4.6 pg/mL/mg of tissue), melittin (26.9 ± 5.0 pg/mL/mg of tissue) or A23187 (29.0 ± 4.6 pg/mL/mg of tissue; Table 1). In contrast, synthesis of PGF2α from explants originating from animals treated with E_2_ was 48.4% greater following exposure to A23817 (43.0 ± 4.2 pg/mL/mg of tissue) compared with explants from control animals (29.0 ± 4.6 pg/mL/mg of tissue; *P ≤* 0.01). Treatment with E_2_*in vivo* did not affect the responses to melittin (26.9 ± 5.0 *vs.* 31.7 ± 4.6 pg/mL/mg of tissue) or OT (28.2 ± 4.6 *vs.* 33.2 ± 4.2 pg/mL/mg of tissue).

**Table 1:** **Concentration of PGF2α (pg/mL/mg of tissue) produced by endometrial explants from cross-bred heifers on Day 17 of the estrous cycle treated *in vivo *** **with 17β-estradiol and *in vitro *** **with A23817, melittin or OT**

**Variable**	**Treatment (mean ± SEM)**			
	**Control**	**E**_**2**_	**Stimulator**	**E**_**2**_**/Stimulator**
DIF60 –A23817	24.7 ± 4.6^b^	20.7 ± 4.2^b^	29.0 ± 4.6^b^	43.0 ± 4.2^a^
DIF60 - melittin	24.7 ± 4.6	20.7 ± 4.2	26.9 ± 5.0	31.7 ± 4.6
DIF60 - OT	24.7 ± 4.6	20.7 ± 4.2	28.2 ± 4.6	33.2 ± 4.2

^a,b^significantly different, *P* ≤ 0.01.

### Experiment 2

**Figure 1:**
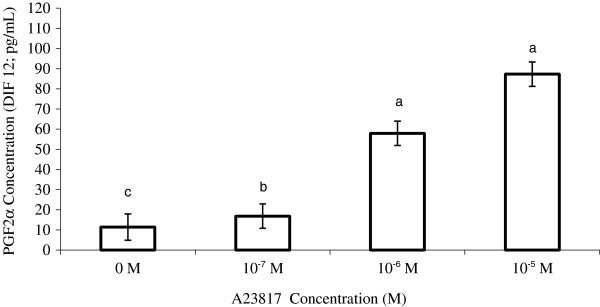
**PGF2α production after 12 h in the culture medium of bovine endometrial (BEND) cells treated with A23817 (DIF12). **^a,b,c^significantly different, *P* < 0.01.

We used A23187 at 10^-7^, 10^-6^ and 10^-5^ mol/L, and these concentrations stimulated dose-dependent increase in PGF2α production (Figure 1). A23187 stimulated the synthesis of PGF2α in all treatment groups compared with that seen in the control group (*P ≤* 0.01). The level of stimulation was greater when A23187 was used at 10^-6^ and 10^-5^ mol/L, in comparison with A23817 at 10^-7^ mol/L (*P ≤* 0.01). We did not observe a significant difference in stimulation levels between 10^-6^ and 10^-5^ mol/L A23817.

### Experiment 3

**Figure 2:**
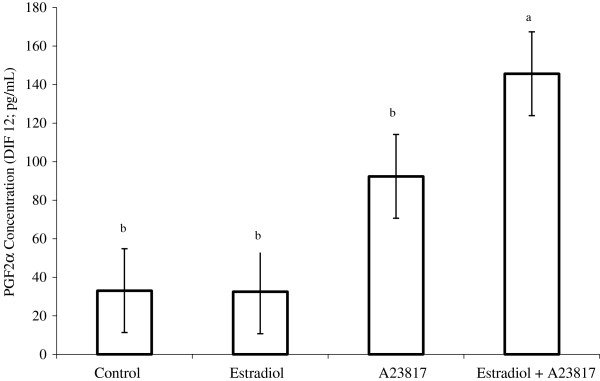
**PGF2α production after 12 h in the culture medium of BEND cells treated with estradiol and/or A23817 (DIF12). **^a,b^significantly different, *P* < 0.01.

Mean PGF2α concentrations were 33.1, 32.5, 92.4 and 145.6 pg/mL (SEM: 21.8 pg/mL) for untreated cells, cells treated with E_2_, cells treated with A23817, and cells treated with E_2_ and A23817, respectively (Figure 2). Production of PGF2α was similar between cells treated with E_2_ and untreated cells. The cells treated with A23817 tended (*P* ≤ 0.08) to produce higher quantities of PGF2α compared with cells treated with E_2_, or cells that were left untreated . The combination of E_2_ and A23817 stimulated greater synthesis of PGF2α compared with the other treatments (*P* < 0.01). A23817 was responsible for a 179% increase in PGF2α production relative to the control group. When A23817 was combined with E_2_, the production of PGF2α increased by 340% compared with that in the control group.

## Discussion

In Experiment 1, PGF2α synthesis was not stimulated in the endometrial explants from cows treated with E_2_*in vivo* and received no treatment *in vitro* (Table 1). These results oppose those reported by Mann [17]. In contrast, Asselin et al. [18] and Xiao et al. [19] reported that the addition of E_2_ to the culture medium was not able to stimulate the release of PGF2α. Because of the inconsistencies in the previously reported data, it was considered appropriate to administer E_2_*in vivo* in this experiment. Previous reports indicated that the administration of E_2_ could stimulate the production of PGFM in cows on days 13 [2,20,21], 17 [20-22], 18 [1,20,23] and 19 [21] of the estrous cycle. Bertan et al. [21] verified that E_2_ promoted an increase in the plasma concentration of PGFM 4 h after injection; its concentration peaked within 6.5 h and returned to basal levels 9 h after treatment. Given this time frame, it was considered appropriate to administer E_2_*in vivo* 2 h prior to euthanizing cows. This procedure allowed the explants to be incubated with different stimulants approximately 6.5 h after the E_2_ injection.

It was expected that E_2_ would stimulate the synthesis of enzymes involved in the synthesis of PGF2α. Thus, E_2_ would increase the concentrations of the corresponding proteins in cellular compartments. It was also expected that the activity of these proteins during PGF2α production would be amplified following specific stimulation. The CI A23817 promotes an increase in intracellular calcium concentration that is responsible for the activation of PKC and PLA2. The stimulant melittin specifically activates PLA2. Melittin and A23817 increase the synthesis of PGF2α in ovine [13,24] and bovine endometrial explants [13,14,17,25,26]; however, this stimulation was not observed in our studies when A23817, melittin or OT was used on their own.

In our Experiment 1, synthesis of PGF2α was observed only when A23817 was used in combination with E_2_. Other researchers observed that the effects of E_2_ were frequently associated with an increase in the concentrations of free calcium in the cytosol [27-29]. We suggest that PKC and PLA2 are present in greater concentrations in the explants from animals treated in vivo with E_2_. Increased production of PGF2α would result in response to this greater concentration of enzymes stimulated by A23817. However, it is possible that higher concentrations of PLA2 in the endometrial explants that were previously exposed to E_2_ could amplify the synthesis of PGF2α in the presence of melittin. This could be explained by the fact that calcium is likely to be the major limiting factor in the activation of PLA2. In summary, we suggest that higher concentrations of PKC and PLA2 were present in the explants treated with E_2_, and that these enzymes promoted an increase in the synthesis of PGF2α in the presence of A23817.

In Experiment 2 (Figure 1), A23817 stimulated the synthesis of PGF2α for all treatment groups. When A23817 was administered at doses of 10^-6^ and 10^-5^ mol/L, stimulation was greater than that when the CI was used at 10^-7^ mol/L. The capacity of BEND cells to synthesize PGF2α [30-32] and the cellular model for the synthesis of PGF2α described by Burns et al. [13] indicate that calcium is responsible for the activation of enzymes such as PKC and PLA2. These enzymes are essential for the synthesis of PGF2α by endometrial cells. Treatment with a CI has stimulated PGF2α synthesis in endometrial explants from sows [33], guinea pigs [34,35], ewes [36-38] and cows [14,15,39].

In our study, CI stimulated PGF2α synthesis in BEND cells in a dose-dependent manner. Other studies have verified the dose-dependent effects of CI on the synthesis of PGF2α in endometrial explants. Lafrance and Goff [40] observed that a CI at 2.6 μg/mL was sufficient to stimulate the synthesis of PGF2α in the endometrial explants of heifers on days 19 or 20 of the estrous cycle. Danet-Desnoyers et al. [14] reported that the biosynthesis of PGF2α stimulated by a CI in the endometrium of cows on day 17 of the estrous cycle exhibited a dose-dependent response. Synthesis was increased by 0, 67 and 107% when doses of 2, 4 and 10 μg/mL were administered, respectively. In the present experiment, doses of 0.052 (10^-7^ mol/L), 0.52 (10^-6^ mol/L), and 5.2 μg/mL (10^-5^ mol/L) increased the production of PGF2α by 46, 105 and 661%, respectively. We propose that lower doses of A23817 stimulate the synthesis of PGF2α in BEND cells, with a significantly amplified response compared with that in endometrial explants. The increase in intracellular calcium concentration is associated with PLA2 activity [41,42] and in the increased availability of AA for the synthesis of PGF2α [41]. Arnold et al. [15] verified that the synthesis of PGF2α was stimulated in bovine endometrium cultured with a CI, regardless of whether it was supplemented with PLA2. Synthesis was greater when the endometrium was treated with a CI and PLA2 compared with explants treated with PLA2 alone. We propose that calcium has an additive effect on the stimulation of PLA2-induced synthesis of PGF2α.

Our hypothesis for Experiment 3 was that E_2_ increases the sensitivity of endometrial cells to calcium. We verified that A23817 promoted an increase of 179% in the production of PGF2α compared with untreated cells. However, when A23817 was used in combination with E_2_, this increase was approximately 340% (Figure 2), similar to our results in Experiment 1. Thus, E_2_ increased the sensitivity of endometrial cells to calcium, supporting the hypothesis of our experiment. Several researchers reported that the synthesis of PGF2α by endometrial explants was not increased by supplementing cultures with E_2_[26,43,44]. Consistently, in Experiment 3, synthesis of PGF2α was not stimulated in BEND cells treated with E_2_ only. The genomic action of E_2_ is promoted by the E_2_-receptor complex, which activates transcription factors that are bound to DNA, ultimately resulting in new protein synthesis [29]. *In vivo*, E_2_ most likely activates the synthesis of PGF2α by stimulating the transcription and translation of proteins involved in the production of PGF2α. However, the activity of E_2_-induced proteins might need further stimulation *in vitro* because of limited access to calcium under culture conditions. In our study, the addition of A23817 to BEND cell cultures increased intracellular calcium concentrations, to enhance intracellular mechanisms that depend on this ion. Therefore, PKC and PLA2, which are involved in the synthesis of PGF2α, might have been activated, to increase synthesis of PGF2α.

In summary, from our experiments, E_2_ did not stimulate the synthesis of PGF2α by endometrial cells and explants cultured *in vitro*. Many other *in vitro* studies have shown that the administration of E_2_ alone in endometrial explants does not stimulate PGF2α synthesis [26,43,44]. We suggest that the participation of E_2_ in the synthesis of PGF2α involves other endocrine and paracrine factors that are absent in cell and explant culture systems. Bertan et al. [21] clearly showed that E_2_ did not immediately affect the cows that were treated on day 17 of the estrous cycle. The interval between the injection of E_2_ and the increase in PGFM serum concentrations suggests that E_2_ acts on the synthesis of PGF2α through a genomic pathway. We speculate that E_2_ directly stimulates the synthesis of proteins involved in PGF2α production; these proteins might include PKC and PLA2, as they contain calcium-dependent activation domains. Further studies are required to test this proposition.

## Competing interests

The authors declare that they have no competing interests.

## Authors’ contributions

CMBM participated in the design of the study, in all experiments and drafted the manuscript. PMC participated in all experiments, FVM participated in *in vitro* experiments, and MB designed and coordinated the study. All authors read and approved the final manuscript.
